# Supplementary material for: Kilometer-scale structure on the core–mantle boundary near Hawaii
Source: Nat Commun. 2022 May 19;13:2787. doi: 10.1038/s41467-022-30502-5 (PMC9120171; doi:10.1038/s41467-022-30502-5)
Supplement: Supplementary file 1 — SUPPLEMENTARY INFO [file 41467_2022_30502_MOESM1_ESM.pdf]

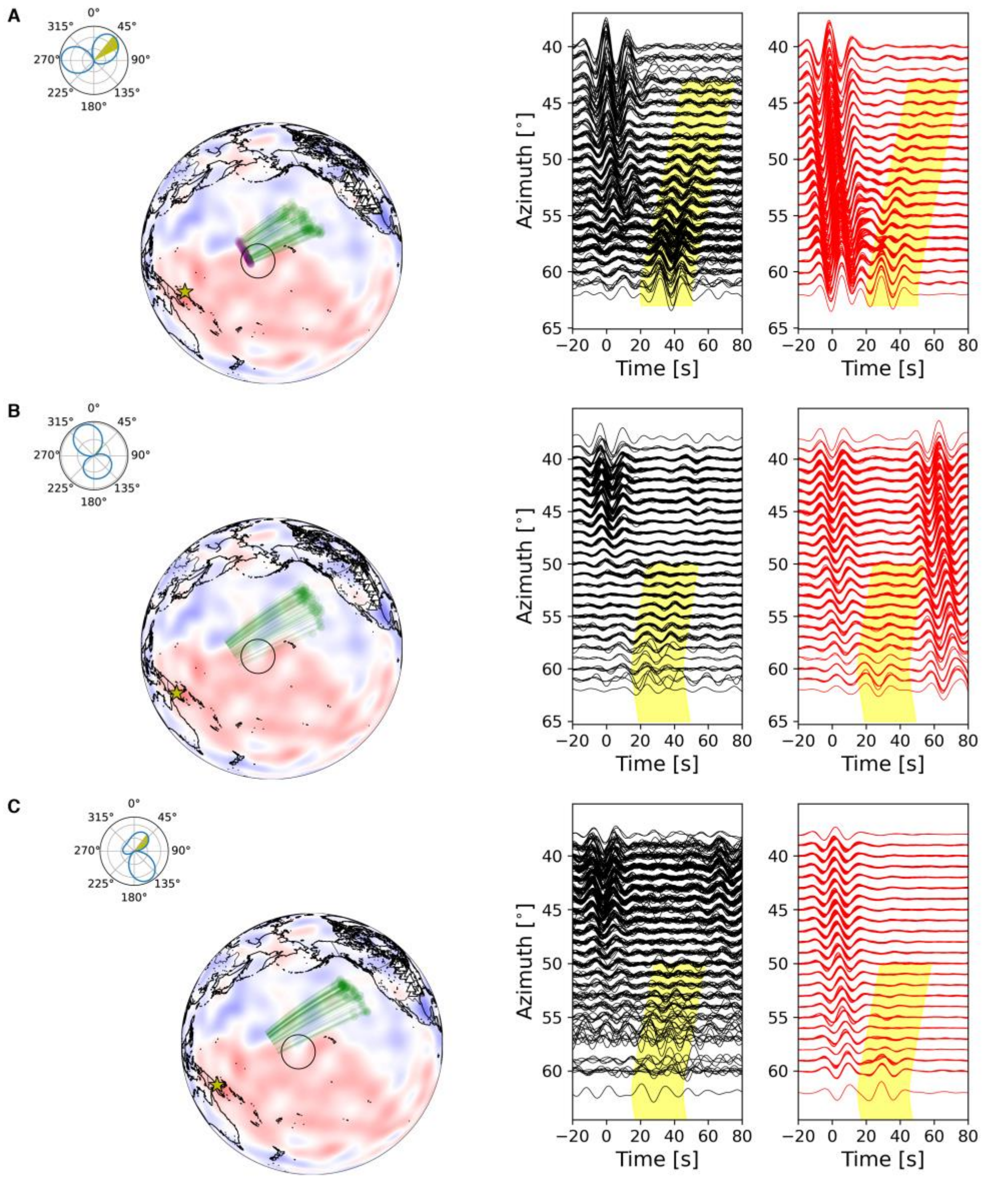

Fig. S1. Papua New Guinea events recorded by the central USarray. For events: (A)20100320 (B)20111214 (C)20120417. On the left we show event radiation patterns (blue) with azimuth range for which data is shown highlighted (yellow). Next is the ray-path geometry shown between the entry (purple) and exit (green) points to the lowermost 100 km of the mantle, the event location (yellow star) and station locations (white triangles). Background shows tomography model SEMUCB_WM1 at 2781 km[38]. The right panels show the observed data of ground motion velocity in the tangential component (black, left) and CSEM synthetics of a 20km -20% $\boldsymbol{dlnV_S}$ ULVZ at the updated location (red, right) filtered from 10s to 20s. The time axis is aligned by the S_{diff} traveltime predicted by the PREM model. Yellow colored bands are sketched by eye and highlight the S_{diff} postcursor.

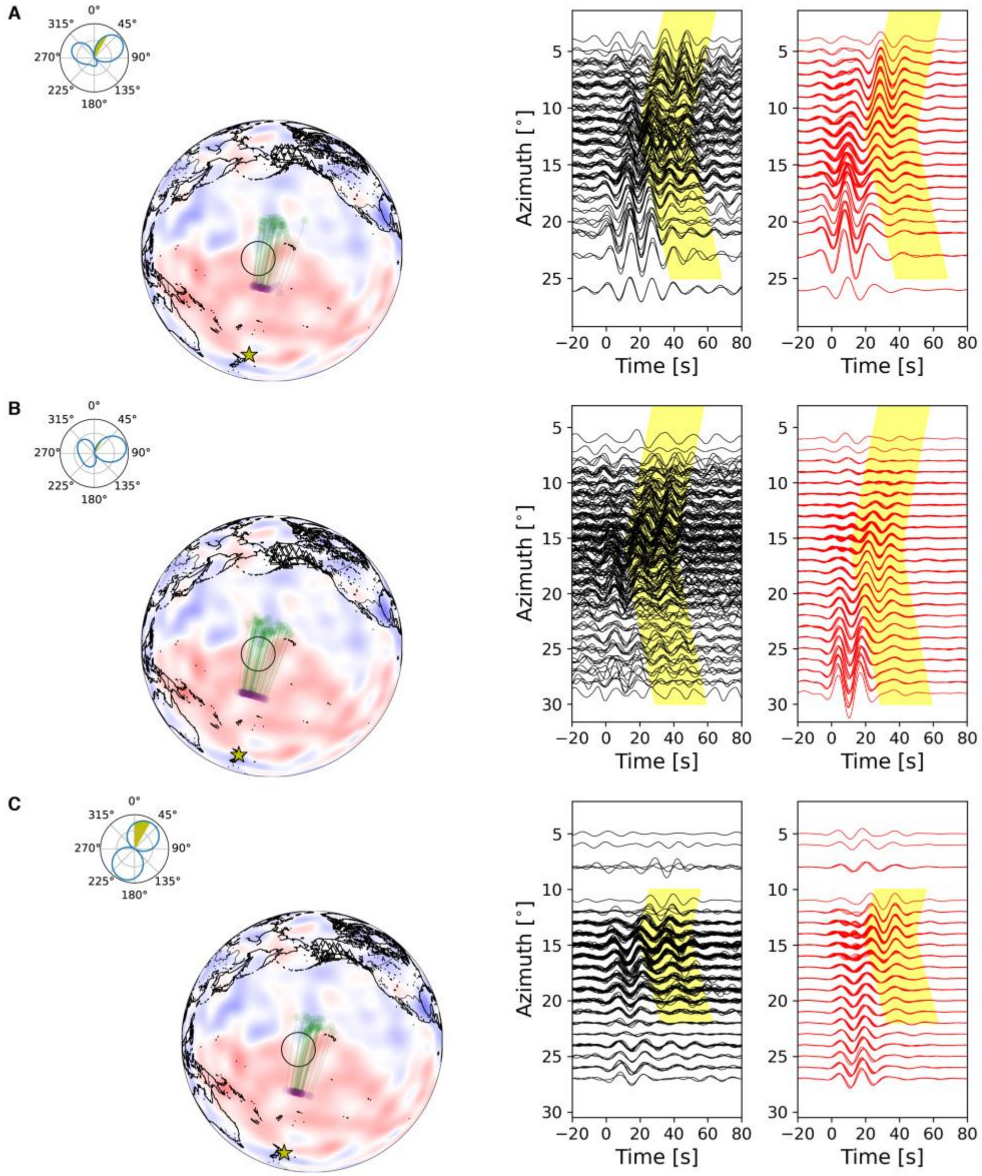

Fig. S2. Same as Fig. S1 but for Kermadec trench events recorded by the Alaska transportable array. For events: (A)20180518 (B)20181030 (C)20161122 (D)20180910

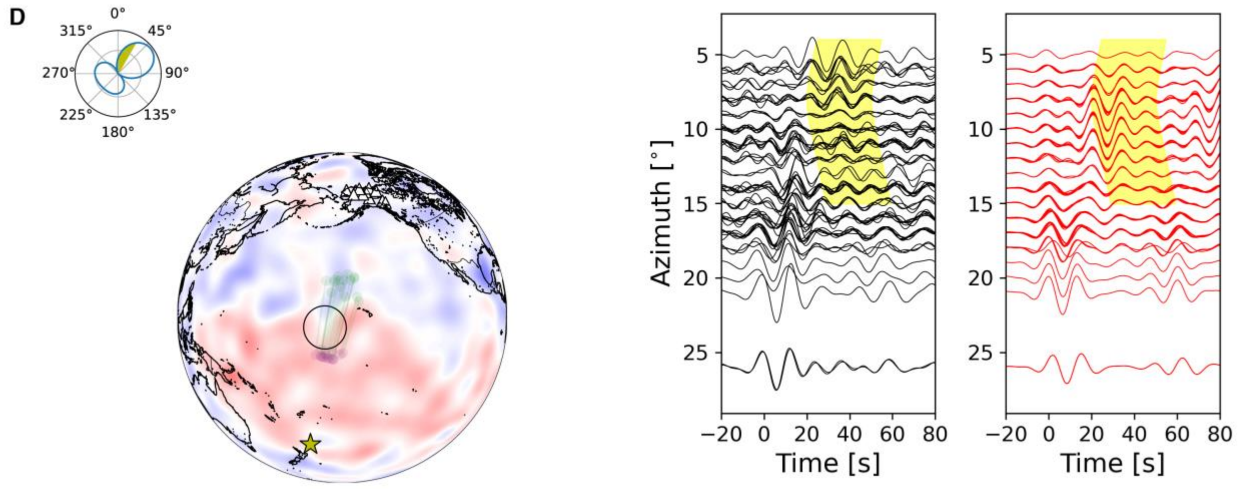
D
0°
45°
90°
135°
180°
225°
270°
315°
Azimuth [°]
5
10
15
20
25
−20
0
20
40
60
80
Time [s]
Time [s]

Fig. S2. (cont.)

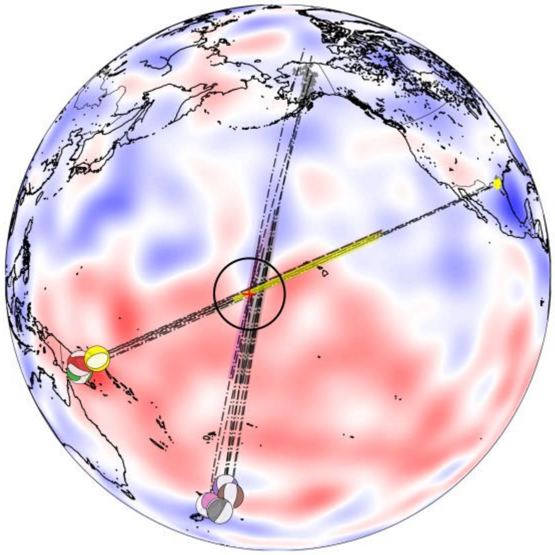

Fig. S3. Paths with the least delayed postcursors for each event intercept at near updated Hawaiian ULVZ location. Azimuth with the least delayed postcursors (shown in black dot dashed lines, 61° azimuth for event 20100320, 61° azimuth for event 20111214, 61° azimuth for event 20120417, 9° azimuth for event 20180910, 11° azimuth for event 20180518, 15° azimuth for event 20181030, 14° azimuth for event 20161122) on the background tomography model SEMUCB_WM1 at 2781 km depth[38]. Beachballs, S_{diff} ray paths (within 1° to the least delayed azimuth), and station notation same as in Fig.1 (including events 20100320 (yellow), 20111214 (green), 20120417 (red), 20180910 (purple), 20180518 (brown), 20181030 (pink), 20161122 (grey), stations (triangles)). Proposed ULVZ location shown with black circle and centred at the red cross (172.3°W, 15.4°N) determined by the intersection of the least postsursor traveltime path in event 20100320 and event 20180910.

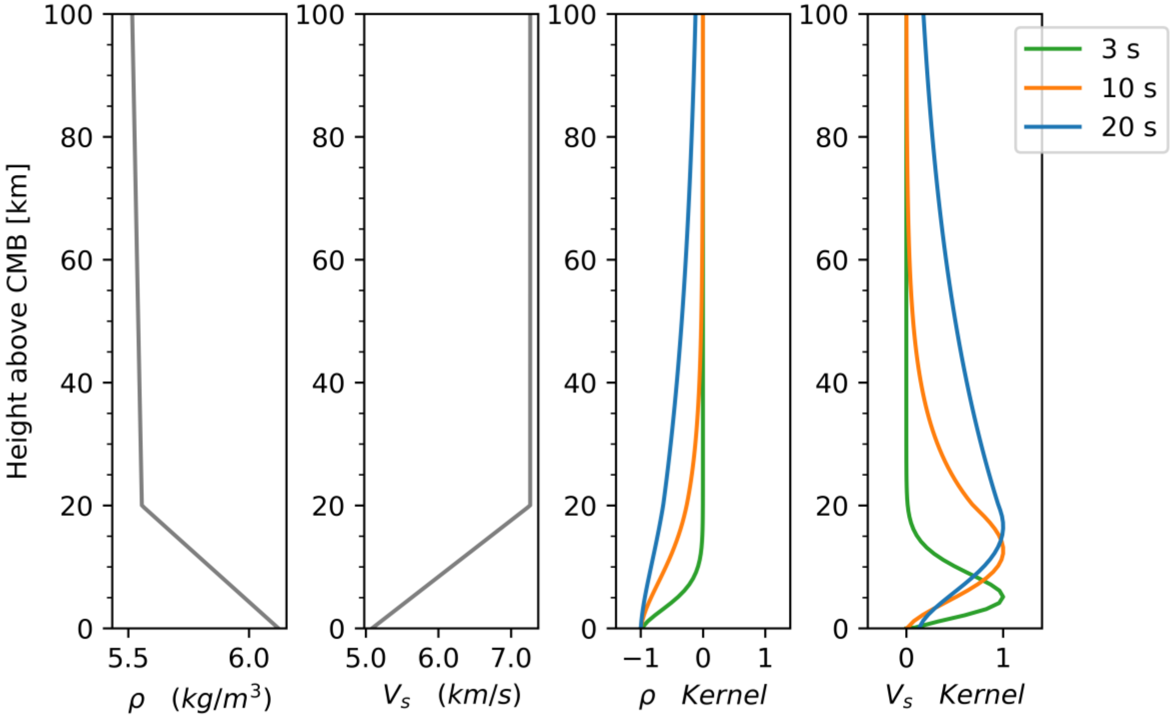

Fig. S4. Estimated sensitivity kernels for S_{diff} wave travel time above the CMB. From left to right: shear wave velocity profile, normalized eigen displacement, traction, and shear wave velocity kernels for a 3s (green), 10s (orange) and 20s (blue) period S_{diff} wave in the bottom 100 km of the mantle.

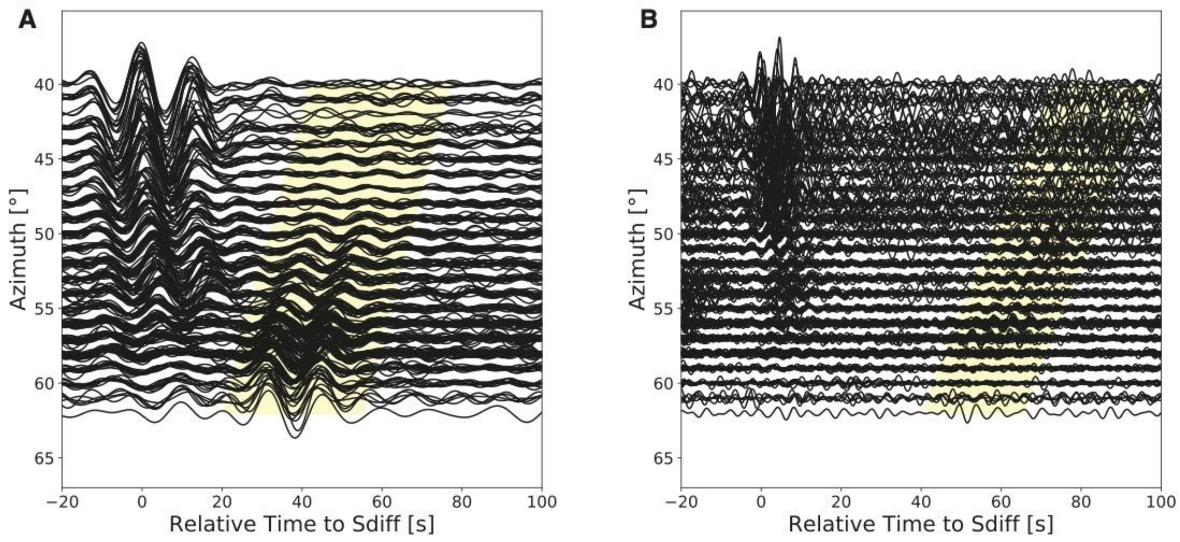

Fig. S5. Original data before stacking. Original data of Event 20100320 at S_{diff} distance (100°-110°) plotted as a function of azimuth, with time axis relative to S_{diff} arrival time predicted by the PREM model. (A) Filtered into long-period range (10s - 20s) and (B) short-period range (3s - 6s). Yellow colored bands highlight the S_{diff} postcursor.

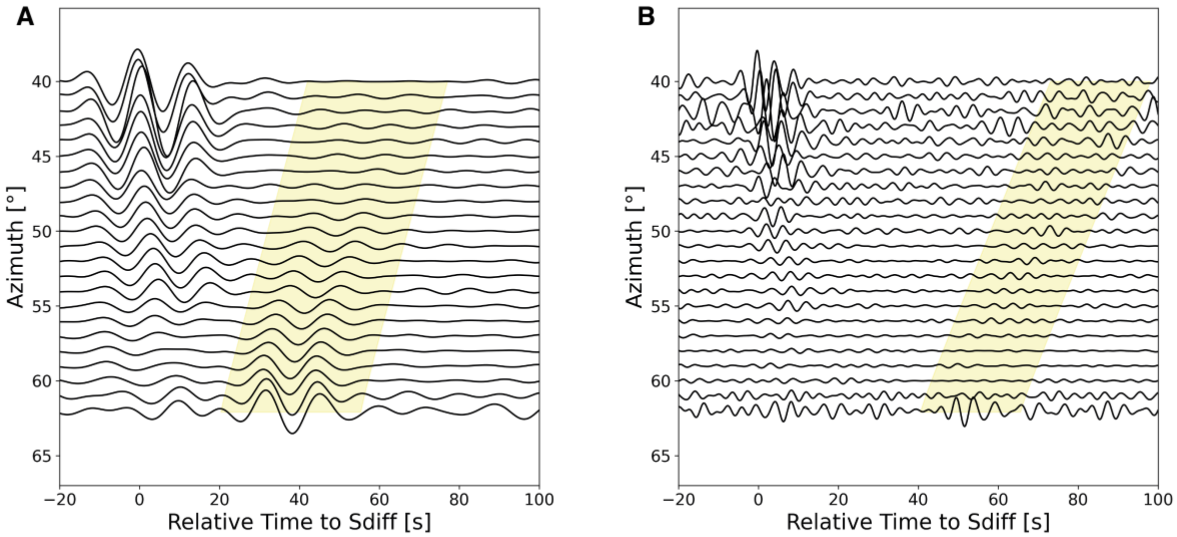

Fig. S6. Linear stack of the waveform based on azimuth bins. Linear stacked data of Event 20100320 at S_{diff} distance (100°-110°) plotted as a function of azimuth, with time axis relative to S_{diff} arrival time predicted by the PREM model. (A) Filtered into long-period range (10s - 20s) and (B) short-period range (3s - 6s). Yellow colored bands highlight the S_{diff} postcursor.

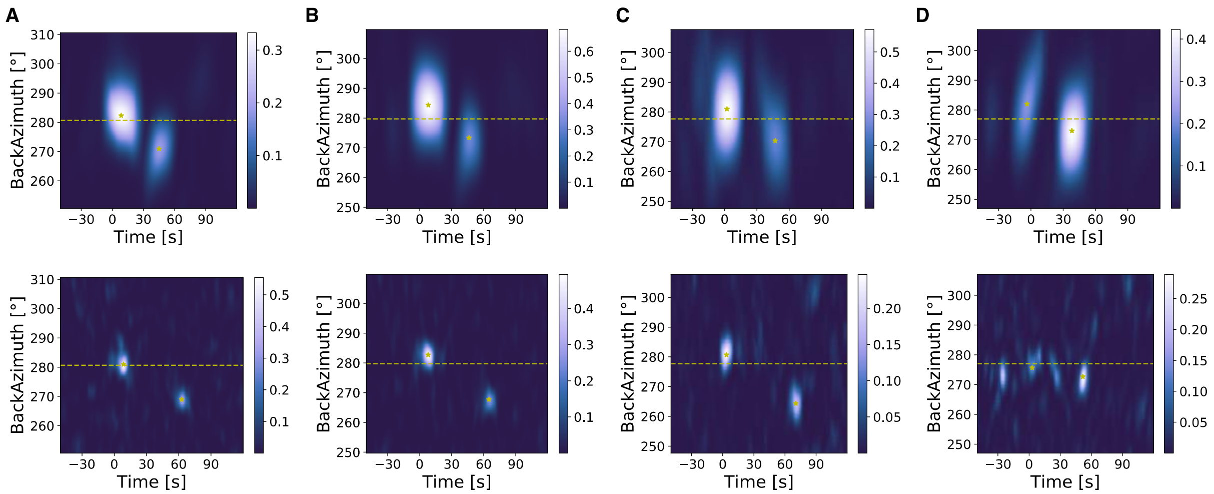

Fig. S7. Beamforming analysis of S_{diff} main arrivals and postcursors of the 20100320 event from four example sub-arrays. (A) Station TUL1 in the transportable array at distance 109.66° and azimuth 52.93° (B) Station U34A in the transportable array at distance 108.19° and azimuth 52.64° (C) Station Q28A in transportable array at distance 104.44° and azimuth 50.46° (D) Station 232A in transportable array at distance 107.10° and azimuth 57.62°. Colors represent the beamforming energy. The yellow dashed line shows the predicted incoming direction. The picked incoming backazimuths and arrival times for the main phase and postcursor are marked with the yellow stars. Top panels show the result for long-period data, bottom panels show the result for short-period data.

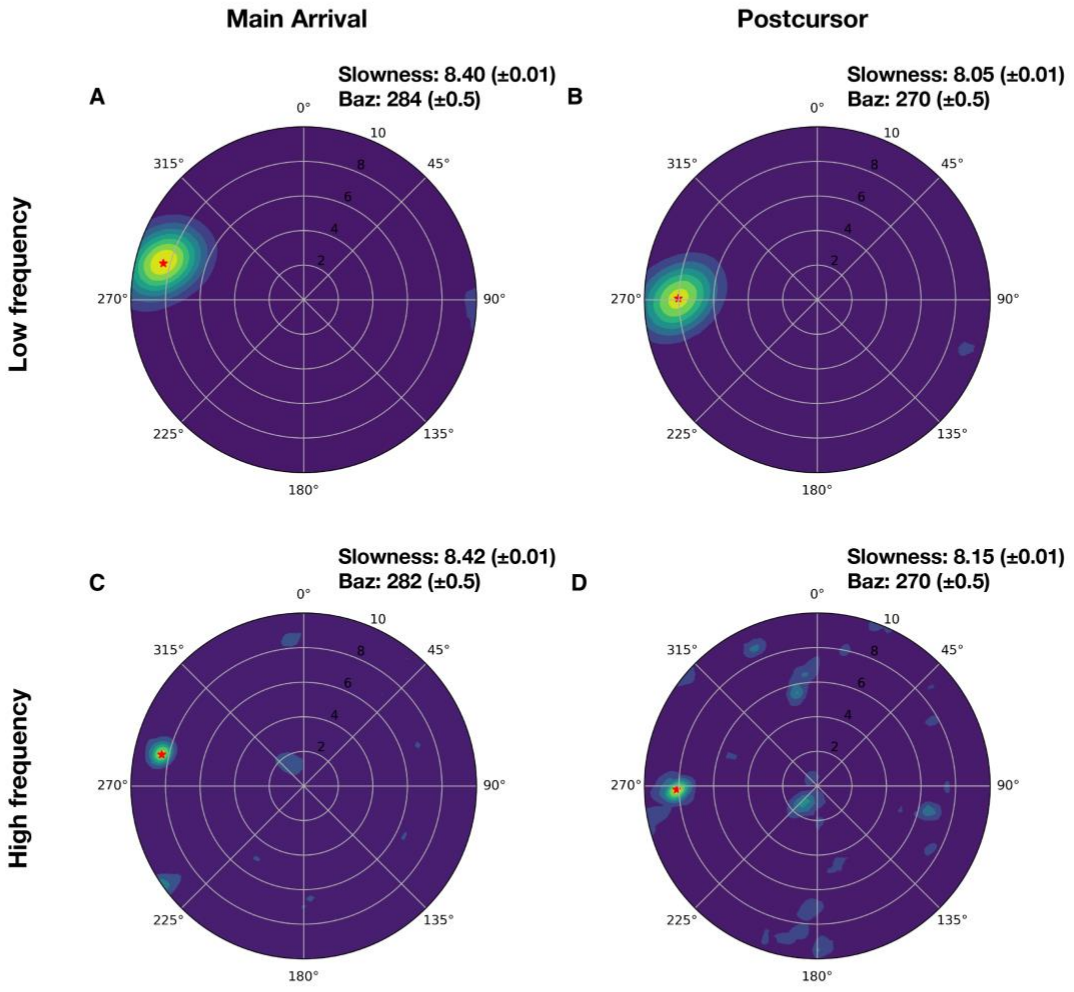

Fig. S8. Sliding-window FK-plot of the Sdiff signal for the subarray around TA.T33A station. (A) Main Arrival in low frequency (Window: -10s - 20s) (B) Postcursor in low frequency (Window: 34s - 64s) (C) Main Arrival in high frequency (Window: -5s - 15s) (D) Postcursor in high frequency (Window: 54s - 74s).

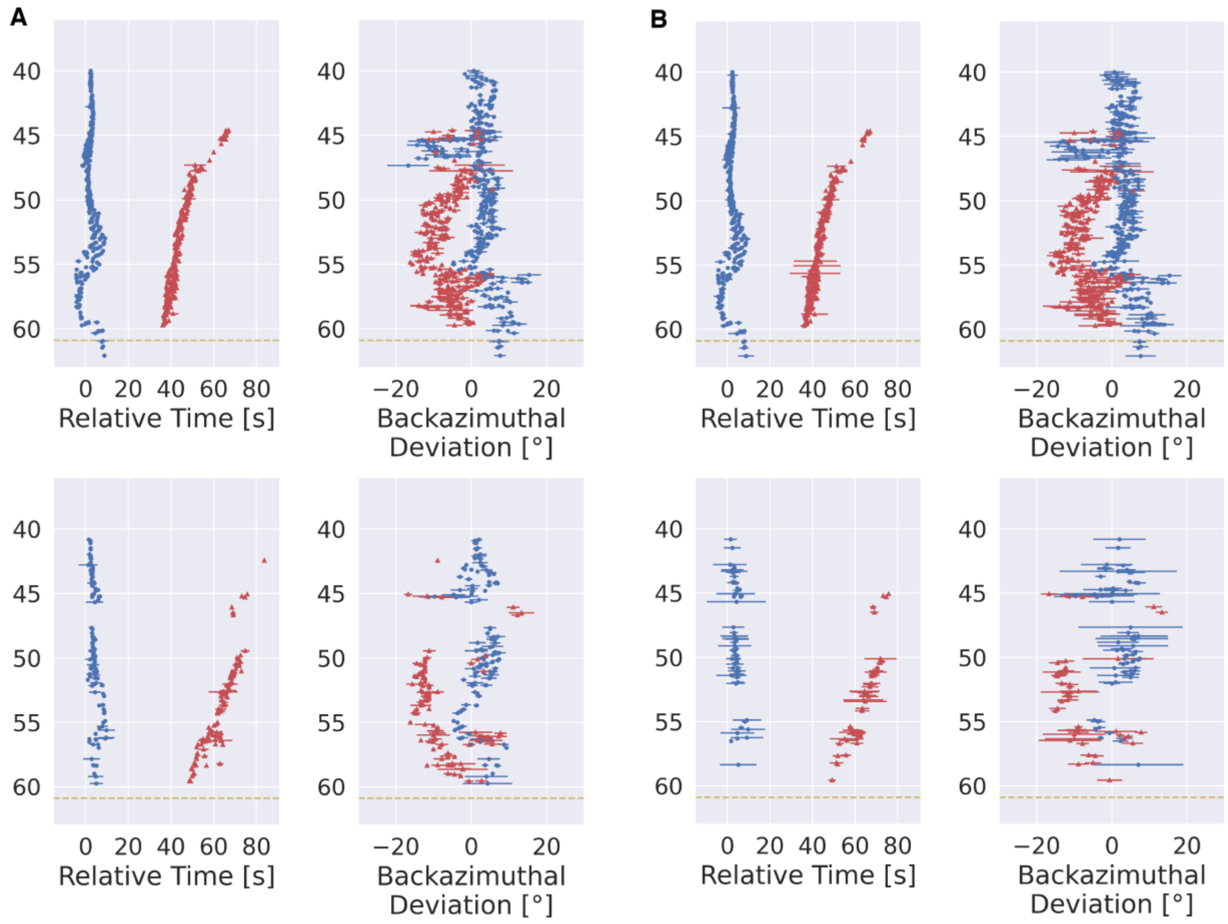

Fig. S9. Robustness comparison of the beamforming result with 5% and 10% slowness variance and 10% slowness variance. (A) Beamforming result of travel time measurements and backazimuth deviations with error bars denoting the max variance of the measurement when the value of Sdiff slowness is varied by ±5%. See Fig. 3 for details (B) Same with A but with error bars denoting the max variance of the measurement when the value of Sdiff slowness is varied by ±10%.

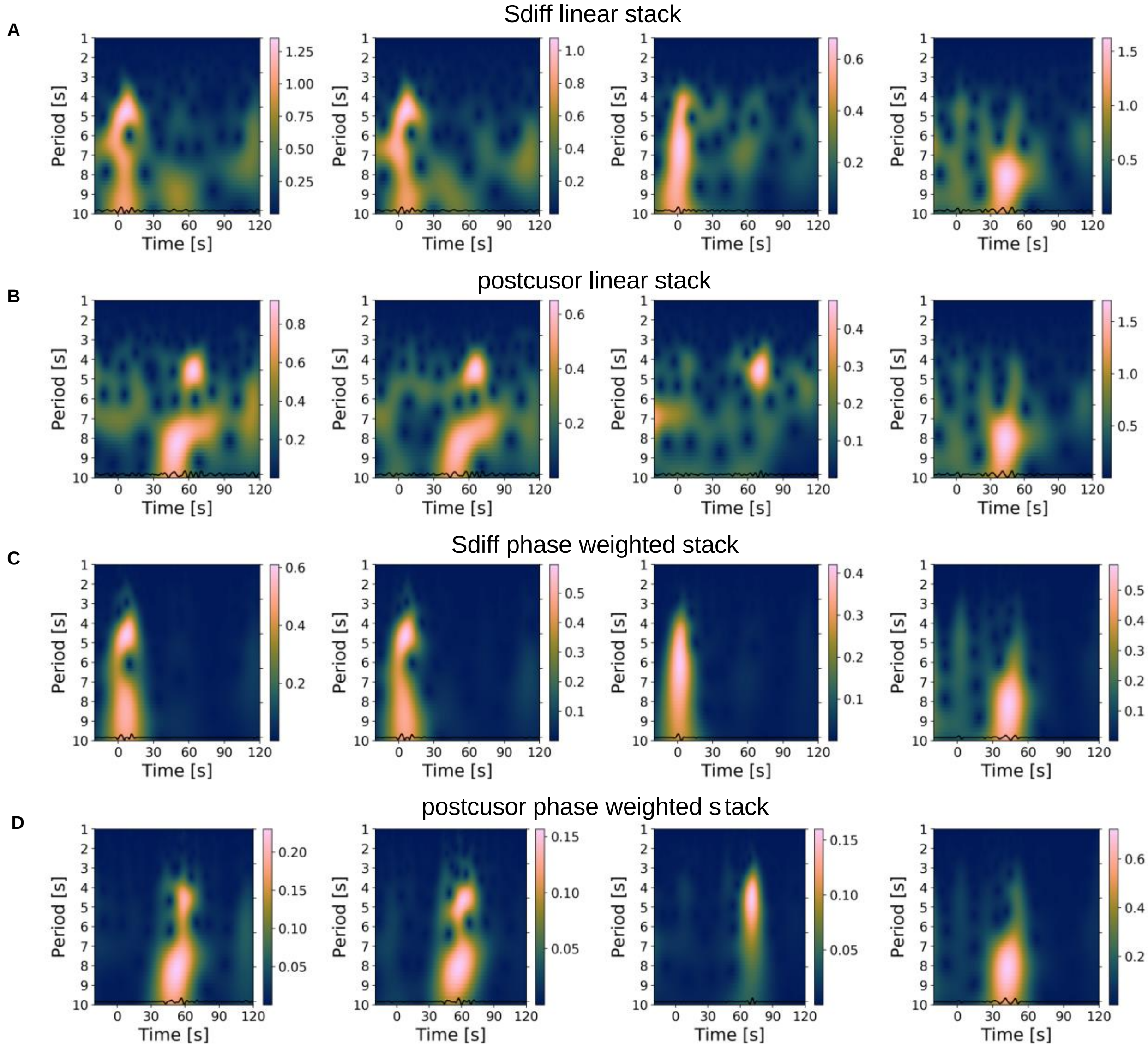

Fig. S10. Stacked wavelet spectrum for the four sample sub-array stacks in Fig.S5 for the 20100320 event. (A) Linear stacked wavelet spectrum using the main arrival incoming directions (B) Linear stacked wavelet spectrum using the postcursor incoming directions (C) Phase-weighted stacked wavelet spectrum using the main arrival incoming directions (D) Phase-weighted stacked wavelet spectrum using the postcursor incoming directions

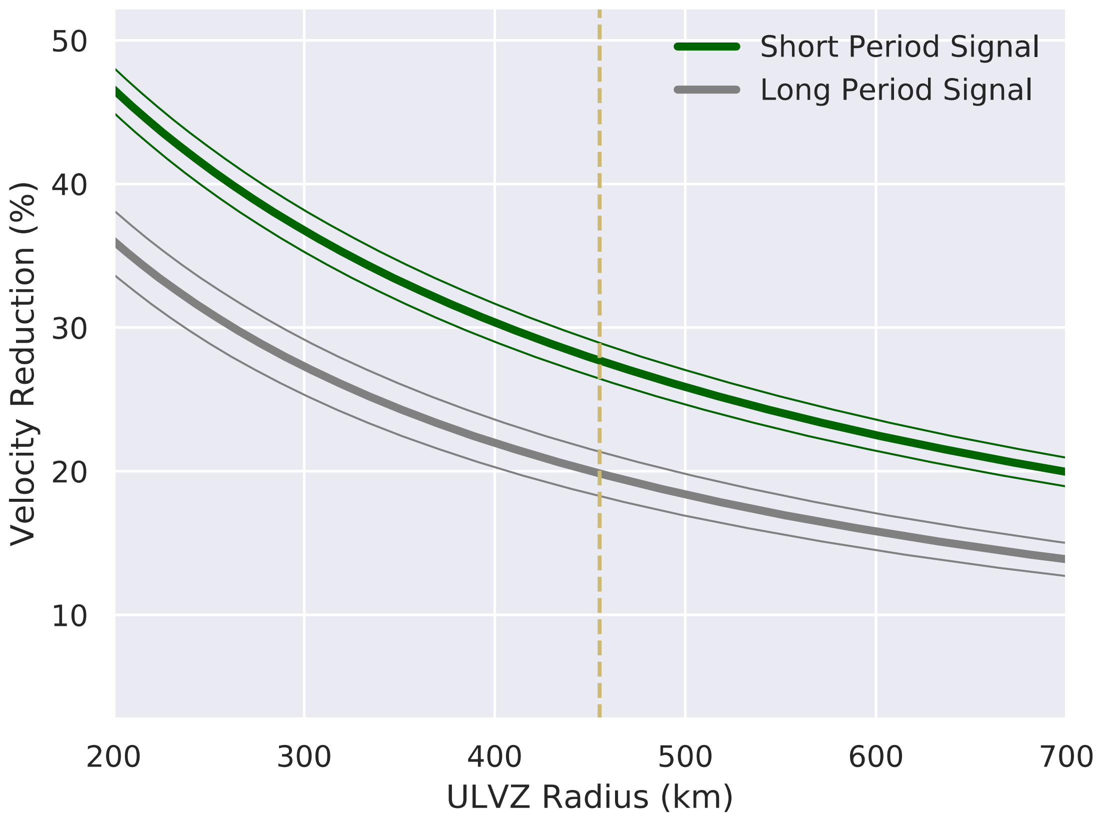

Fig. S11. Trade-offs between velocity reduction and ULVZ radius. This calculation is based on a simple cylindrical ULVZ structure and ray-theoretical assumptions. The green line indicates the tradeoff based on the observed short-period postcursor arrival time, while the gray line shows the long-period tradeoff. The thin line marks an uncertainty of ±3s. The vertical yellow dashed line shows the radius of the favored ULVZ model in the study of [7].

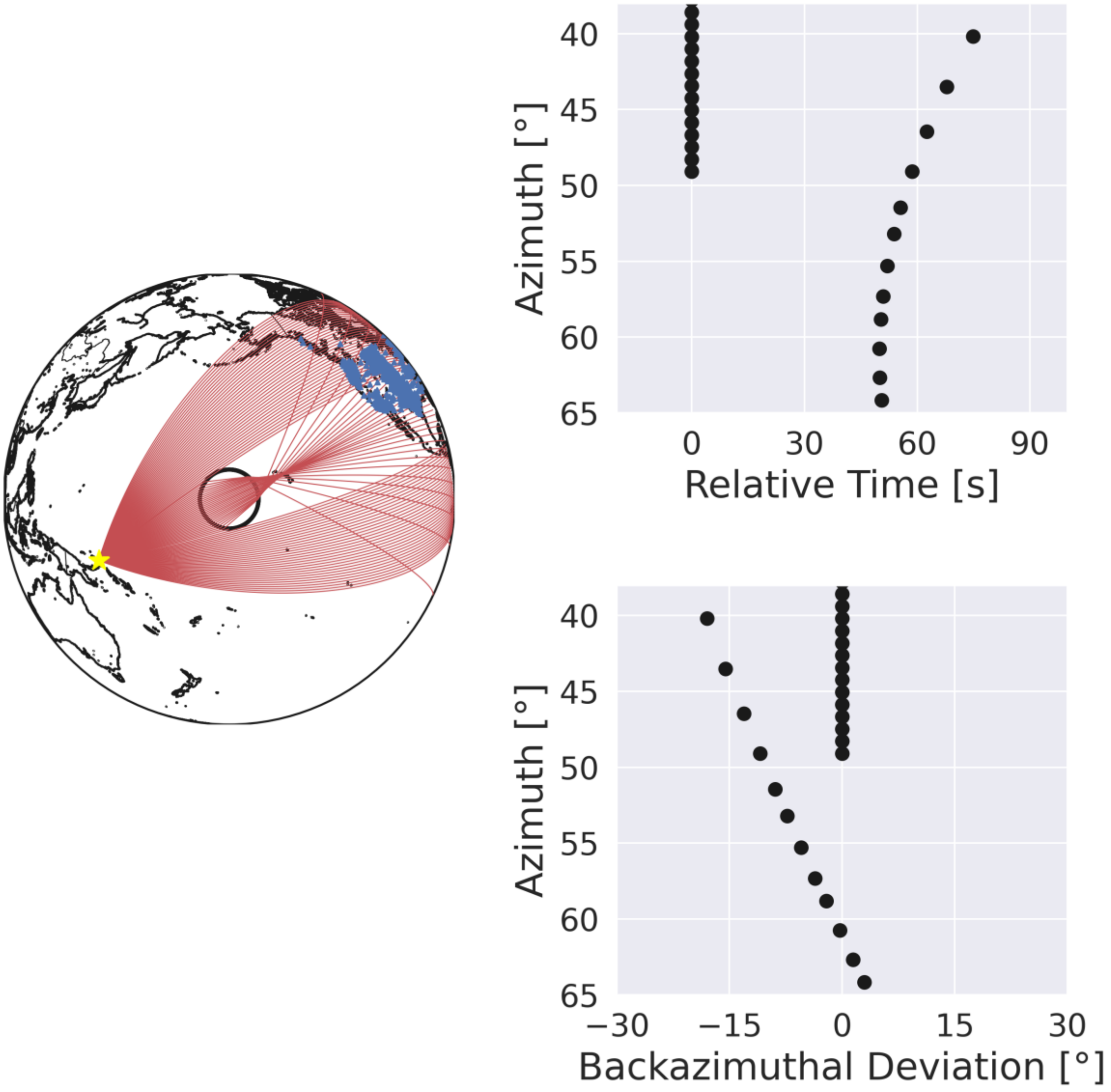

Fig. S12. Ray-based modelling of travel-time and backazimuth. The left shows the ray geometry focused by a cylindrical ULVZ. The upper right shows the travel-times of the rays as a function of the azimuth at the receiving station. The lower right shows the backazimuth deviation of the rays concentrated by the ULVZ structure of a 455km radius and 40% vs reduction.

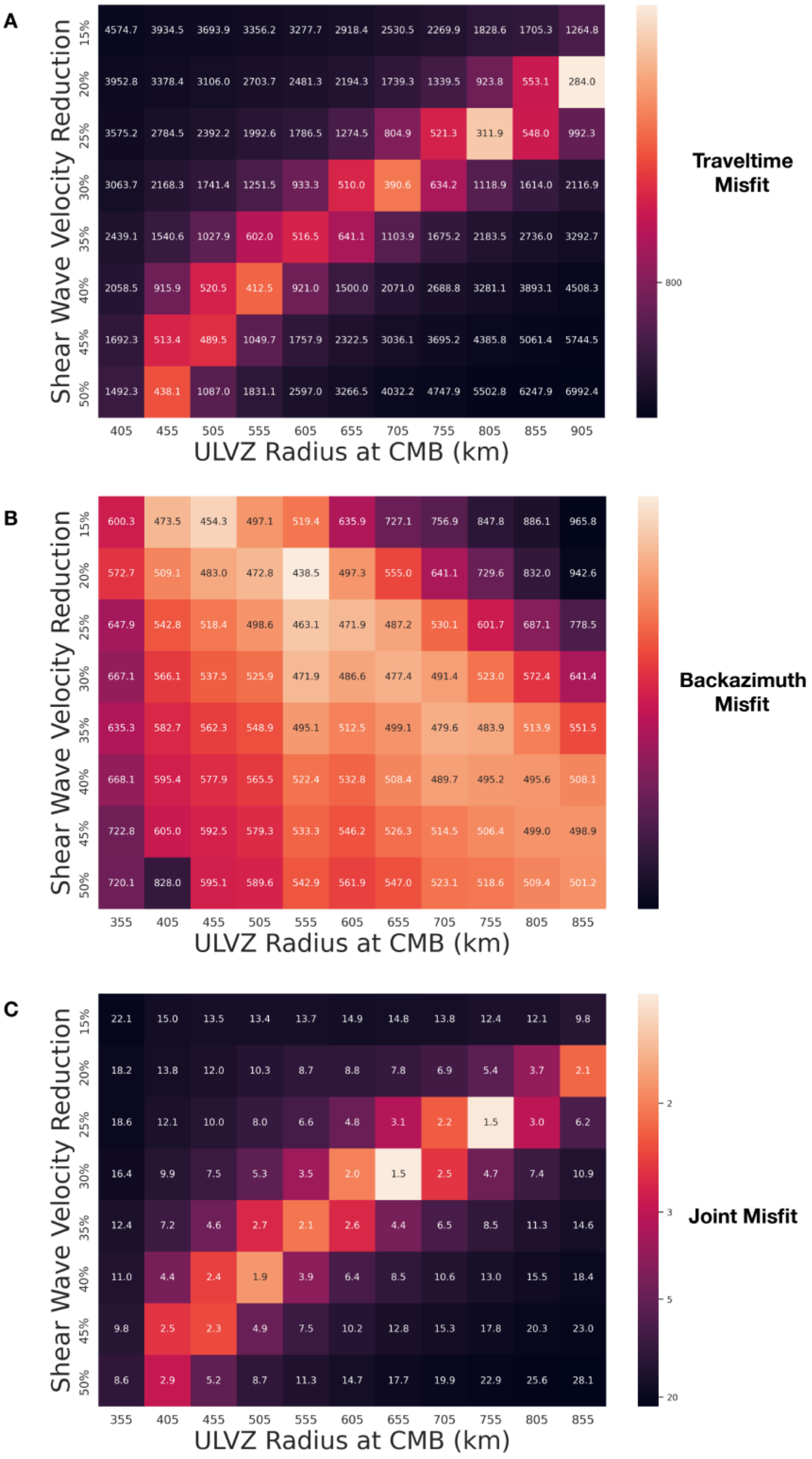

Fig. S13. Misfit colormap of the short-period data for ULVZ models of varying radius and velocity reduction calculated using raytracing. For misfits based on: (A) postcursor arrival time (B) postcursor backazimuth (C) joint misfit for arrival time and backazimuth.

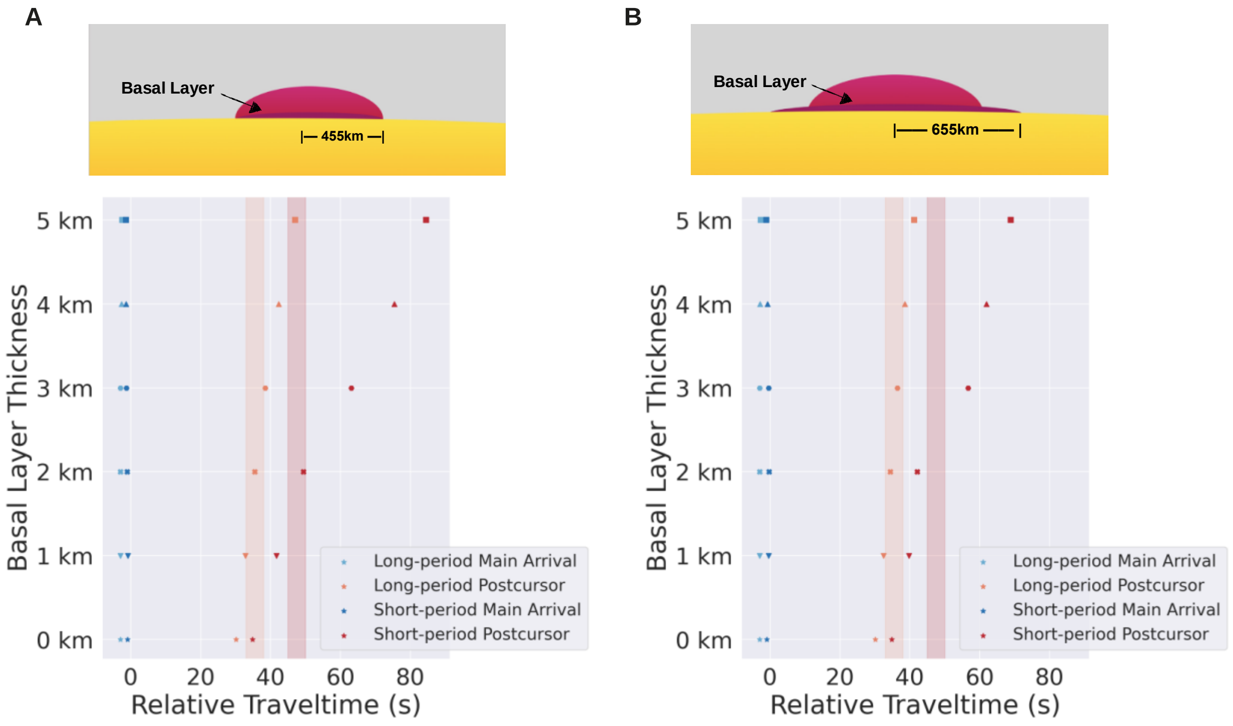

Fig. S14. Observed travel-times for postcursors 2.5D AxiSEM synthetics for two-layered ULVZ models with different basal layer thickness. The faded color bands show the observed arrival time of long-period (orange) and short-period (red) postcursors (A) Two-layered models with constant 455km radius. The bottom - 40% $dlnV_S$-layer varies from 0 to 5km thick below a total thickness 20km -20% $dlnV_S$ layer. (B) Same as (A) but with a wider bottom layer of 655km radius.

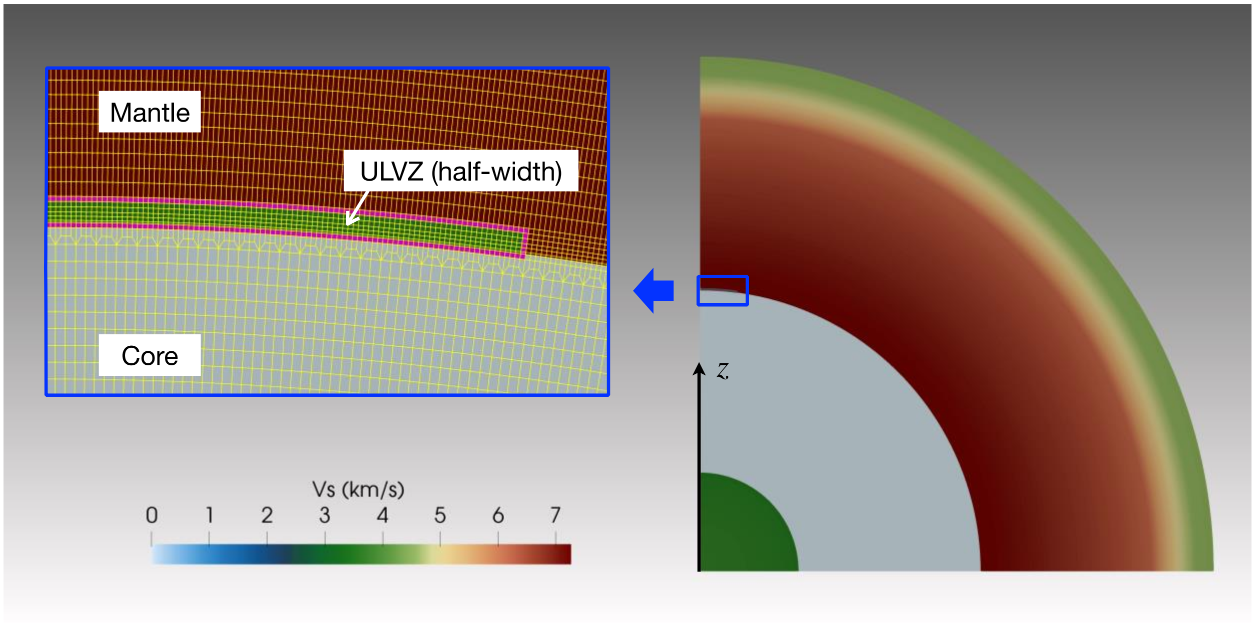

Fig. S15. AxiSEM3D mesh used in the modeling. On the right, we show the 2D AxiSEM3D mesh (gridlines and southern half hidden) created using the PREM model[37] at a 3 s period. On the left, we zoom into the blue box atop CMB (gridlines revealed), which includes the half-width ULVZ (7.5° on CMB) with a height of 20 km. This mesh is centred at the ULVZ, rotating which by 360° about the left axis will generate the 3D ULVZ structure, spanning 15° on CMB. The 3D scattered wavefield is excited by injecting an incident wavefield simulated with a PREM mesh (no ULVZ) centred at the earthquake source and recorded at the "injection elements" around the ULVZ (the elements coloured in magenta in the mesh)[20]

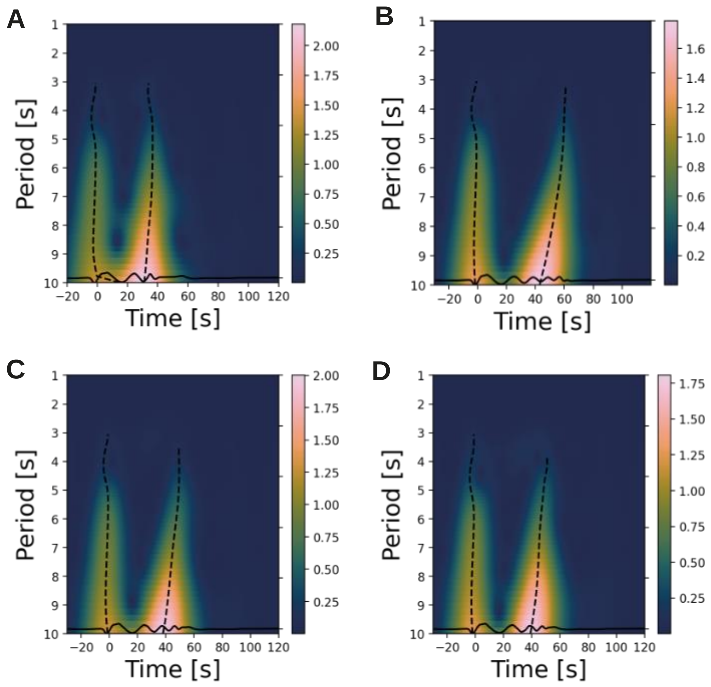

Fig. S16. Wavelet spectra of single synthetic waveforms for four 3D ULVZ models sampling the center of the ULVZ. (A) the uniform model (B) the two-layer model (C) the gradient model (D) the extreme 2km model. These spectra can be compared to the real data stacks in Fig. S10.
